# Supplementary material for: A Critical View of the Application of the APEX Software (Aqueous Photochemistry of Environmentally-Occurring Xenobiotics) to Predict Photoreaction Kinetics in Surface Freshwaters
Source: Molecules. 2019 Dec 18;25(1):9. doi: 10.3390/molecules25010009 (PMC7017383; doi:10.3390/molecules25010009)
Supplement: Supplementary file 1 [file molecules-25-00009-s001.zip › 3.2.4_gcc-4.4.0.zip!/3.2.4_gcc-4.4.0/3.2.4_gcc-4.4.0/doc/octave/html/interpreter/Variable_002dlength-Argument-Lists.html]

Variable-length Argument Lists - Untitled


Next: Variable-length Return Lists,
Previous: Multiple Return Values,
Up: Functions and Scripts


---

### 11.3 Variable-length Argument Lists

Sometimes the number of input arguments is not known when the function
is defined. As an example think of a function that returns the smallest
of all its input arguments. For example,

```
     a = smallest (1, 2, 3);
     b = smallest (1, 2, 3, 4);
```

In this example both `a` and `b` would be 1. One way to write
the `smallest` function is

```
     function val = smallest (arg1, arg2, arg3, arg4, arg5)
       body
     endfunction
```

and then use the value of `nargin` to determine which of the input
arguments should be considered. The problem with this approach is
that it can only handle a limited number of input arguments.

If the special parameter name `varargin` appears at the end of a
function parameter list it indicates that the function takes a variable
number of input arguments. Using `varargin` the function
looks like this

```
     function val = smallest (varargin)
       body
     endfunction
```

In the function body the input arguments can be accessed through the
variable `varargin`. This variable is a cell array containing
all the input arguments. See Cell Arrays, for details on working
with cell arrays. The `smallest` function can now be defined
like this

```
     function val = smallest (varargin)
       val = min ([varargin{:}]);
     endfunction
```

This implementation handles any number of input arguments, but it's also
a very simple solution to the problem.

A slightly more complex example of `varargin` is a function
`print_arguments` that prints all input arguments. Such a function
can be defined like this

```
     function print_arguments (varargin)
       for i = 1:length (varargin)
         printf ("Input argument %d: ", i);
         disp (varargin{i});
       endfor
     endfunction
```

This function produces output like this

```
     print_arguments (1, "two", 3);
          -| Input argument 1:  1
          -| Input argument 2: two
          -| Input argument 3:  3
```

— Function File: [reg, prop] = **parseparams** (params)  
> Return in reg the cell elements of param up to the first
> string element and in prop all remaining elements beginning
> with the first string element. For example
>
> ```
>           [reg, prop] = parseparams ({1, 2, "linewidth", 10})
>           reg =
>           {
>             [1,1] = 1
>             [1,2] = 2
>           }
>           prop =
>           {
>             [1,1] = linewidth
>             [1,2] = 10
>           }
> ```
>
> The parseparams function may be used to separate 'regular'
> arguments and additional arguments given as property/value pairs of
> the varargin cell array.
>
>
>
> **See also:** varargin.
